# P07 Finding recovery, trust and hope in silence. Psychological treatment in chronic pain

**DOI:** 10.1093/rap/rkac067.007

**Published:** 2022-09-28

**Authors:** Neelo Aslam, Tessa Hutton

**Affiliations:** Royal Manchester Children's Hospital, Manchester, United Kingdom; Royal Manchester Children's Hospital, Manchester, United Kingdom

## Abstract

**Introduction/Background:**

This case has many important discussions and demonstrates the role of psychological therapy in treatment. The young person presented with chronic pain and was unable to walk (in a wheelchair). There had been a missed ASD diagnosis due to atypical presentation. There were accusations of fabricated illness which had led to mistrust of professionals and had impacted on family. The patient’s selective mutism was a challenge to therapeutic approach and required increased creativity and flexibility. This case highlights the impact of Sensory processing difference and the relationship to chronic pain. The patient’s significant anxiety led to treatment limitations.

**Description/Method:**

This young person was referred to us after they had been seen by several teams over a significant period. There were multiple presenting issues without a systemic understanding of the problems. It was important to review previous assessments and information thoroughly and have transparent discussions with the family. The two most difficult tasks were gaining the families trust and to engage a young person who did not speak. The previous MDT had raised safeguarding concerns, in particular the possibility of fabricated illness; this was based on the conclusion that mothers account of social communication difficulties differed from that of school. There had also been concerns raised about the young person’s engagement with professionals and difficultly developing relationships.

**Discussion/Results:**

We approached the case with an open mind. We reassessed and made a diagnosis of ASD. The MDT approach to care is very important and has many positives however it can also lead to “groupthink” and make it difficult to challenge the group view. This helped us and the family to understand the evolution of presenting symptoms and for them to begin to feel believed. Common disorders can present in unusual ways, and this is particularly true for developmental disorders such as ASD.

Sensory processing differences are part of ASD and impacted significantly on the symptoms of pain, loss of function and the ability to engage the young person in physical therapy. The patients difficulty with change and rigidity of thought needed care consideration when supporting engagement with new aspects of care. It also prompted us to think about sensory processing differences and how they may be linked to the presentation of chronic pain and use this understanding to promote engagement with treatment.

This young person was selectively mute and we used novel strategies to engage them in psychotherapy – raising the issue of engaging someone who doesn’t talk in talking therapies. We utilised activities and their interests to develop a therapeutic relationship and motivate engagement in recovery. Given the levels of mistrust and anxiety this took a significant amount of time.

It was an essential task to repair the relationships with key agencies and educate them about this child’s needs. In complex cases this systemic working is essential to ensure progress is made and maintained.

**Key learning points/Conclusion:**

Listen to families

Look at the evidence critically and question your clinical hypothesis.

Don’t be afraid to question and challenge the MDT – this should be a part of a functional MDT.

Case management is an important part of care and recovery.

Continued MDT communication and supervision maintains hope and manages the psychological burden of care.

Work with the patient’s interests and strengths and utilise this to motivate recovery.

Building trust can take time – be patient and consistent.

